# Assessing the Transitional Impact and Mental Health Consequences of the COVID-19 Pandemic Onset

**DOI:** 10.3389/fpsyg.2020.607976

**Published:** 2021-01-08

**Authors:** Eamin Z. Heanoy, Liangzi Shi, Norman R. Brown

**Affiliations:** ^1^Department of Psychology, University of Alberta, Edmonton, AB, Canada; ^2^Department of Psychology, College of New Caledonia, Prince George, BC, Canada

**Keywords:** COVID-19, depression, anxiety, stress, mental well-being, transition theory

## Abstract

In this article, we report the results of a survey of North American adults (*n* = 1,215) conducted between March 24 and 30, 2020 at the onset of the COVID-19 pandemic. Respondents completed the COVID-TIS (Transitional Impact Scale-Pandemic version) and the 21-item Depression, Anxiety, and Stress Scale (DASS), indicated their level of COVID-infection concern for themselves and close others, and provided demographic information. The results indicated: (a) during its early stage, the pandemic produced only moderate levels of material and psychological change; (b) the pandemic produced mild to moderate levels of psychological distress; (c) respondents who lost their jobs as a result of the pandemic experienced more change and more psychological distress than those who did not, and (d) younger respondents and less well-educated ones experienced more psychological distress than older respondents. Unexpectedly, (e) respondents indicated that they were more concerned that friends and family members would become infected with COVID-19 than that they would be. We conclude by speculating that these results are driven less by the immediate changes brought about by the pandemic and more by uncertainty concerning its long-term economic and social impact.

## Introduction

In this article, we report the results of a survey conducted between March 24 and 30, 2020, less than 2 weeks after the World Health Organization (WHO) officially labeled the COVID-19 as a pandemic on March 11, 2020, and within 3 days of the United States (US)–Canada border being closed to non-essential travel on March 21, 2020 (World Health Organization, 2020). This was also about the time, in North America, when learning and office work was moving on-line, retail establishments were closing, and the pandemic was coming to dominate the news cycle and social interactions. At that time, it was already clear that the pandemic was changing peoples’ lives. What was less clear was *how* the pandemic was changing those lives. Thus, our survey was designed to assess: (a) the extent and nature of the changes caused by the pandemic, (b) the effect of the pandemic on people’s mental health, and (c) the degree to which the two were related. We were also interested in understanding, at that early stage in the pandemic, (d) how concerned people were with becoming infected with the coronavirus and whether they were more concerned for themselves than they were for their family and friends.

The project takes Transition Theory (Brown et al., 2012, 2016; Brown, 2016; Brown, unpublished) as its starting point. According to this theory, a transition is an event or series of events that causes fundamental changes in the “the fabric of daily life” – what people do, where they do it, and with whom. In addition to affecting their material circumstances, major life transitions also influence people’s behavior, their mental states (e.g., their attitude, thoughts, and sense of self), and their physical and emotional well-being (Holmes and Rahe, 1967; Wyler et al., 1971; Sarason et al., 1978; Wheaton, 1990; Turner and Wheaton, 1995; Rutter, 1996; Tennant, 2002; Svob, et al., 2014). From this perspective, the pandemic, even during its early stages, could be seen as a potentially very important, possibly the largest collective transition, one that needed to be documented from its start and followed as it evolved.

In the past few years, researchers in the field have been using Transitional Impact Scale (TIS-12, Svob et al., 2014; Nourkova and Brown, 2015; Shi and Brown, 2016; Gu et al., 2017; Uzer, 2020; Uzer et al., 2020) to measure the impact of candidate transitional events on people’s lives. TIS consists of 12 items, evaluating material and psychological impacts separately. In response to each item, such as “This event has changed the activities I engage in,” participants rated their agreement on a five-point Likert scale. Theoretically, an event that scores higher than three (neutral) would indicate at least a moderate life impact. More generally, major transitions, i.e., ones that have been found to define important lifetime periods, elicit TIS scores of 4.0 or above (Nourkova and Brown, 2015; Uzer and Brown, 2015; Gu et al., 2017; Uzer et al., 2020; Uzer, 2020).

To measure transitional impacts, we need to also consider individual differences. For example, starting university might be a more impactful transition for “dormies” (university students who left home and came to live in a university dormitory) than for “homies” (university students still living with their parents). Relocation might be a more influential transition for people immigrating from one country to another than those who are relocating from one city to another city within the same state or province. Likewise, we expected that the pandemic would affect some people more than others. Specifically, one of the striking aspects of the pandemic, in its early phase, was widespread job loss. It seemed reasonable to expect that people who had lost their jobs would, on average, experience greater COVID-related change than those who had not and that this would be reflected in higher TIS ratings. We note that the data reported in this article were collected over the web from a large convenience sample (*n* = 1,215). It turned out that a relatively large number of respondents (*n* = 187; 15.4%) indicated that they had lost their jobs as a direct result of the pandemic. This made it possible to test the prediction that job loss would amplify the (negative) effects of the pandemic (Cobb and Kasl, 1977; Dooley and Catalano, 1980; Caplan et al., 1989).

In the present study, we intended to determine how the pandemic was affecting people’s lives during its early stage. Intuitively, we expected that individuals, at least those who had not lost their jobs, would not produce high TIS scores for material change because the pandemic appeared to have altered their lives by narrowing them – by limiting what they could do and where they could do it. We had no firm prediction concerning the responses to the TIS questions used to assess the psychological impact of the pandemic. On the one hand, prior research had found that material change and psychological change were often positively correlated (Holmes and Rahe, 1967; Wyler et al., 1971; Sarason et al., 1978; Turner and Wheaton, 1995; Svob et al., 2014; Gu et al., 2017). On the other hand, the pandemic appears to be unprecedented in its scope and in the ways that societies have reacted to it (e.g., lockdowns, self-isolation, crashing financial markets, and historically high levels of unemployment). It seemed possible that people may have responded to these exceptional times by revising their beliefs about the world and themselves. If so, we should expect at least moderate levels of psychological change.

Prior studies have shown that major life transitions have a strong effect on mental health (Holmes and Rahe, 1967; Wheaton, 1990; Rutter, 1996; Tennant, 2002). During the pandemic, people were already facing economic uncertainty, fear of infection, social isolation, and school- and work-related disruptions, and that these issues are related to negative mental health outcomes (Fitzpatrick et al., 2020; Tull et al., 2020; Zandifar and Badrfam, 2020). Therefore, we anticipated that relatively high levels of depression, anxiety, and stress would be reported in our sample, especially from those whose lives were directly impacted by the pandemic (i.e., job loss).

In addition to the transitional impact of the pandemic and its effect on mental health, we were also interested in how concerned people were that they would be infected by the coronavirus and how concerned they were that others they know might be. We included infection-concerns questions to gauge the level of COVID-specific fear in our sample and to determine whether this form of fear was related to the psychological change experienced by our respondents and to their current levels of depression, anxiety, and stress.

To sum up, we measured the transitional impact of the COVID-19 pandemic, its relation to mental health, and people’s concerns as functions of job status, age, and education. Job-status (job loss vs. no loss) served as a fixed factor in all the analyses. We selected age and education as covariates because older adults were the group at risk for COVID-19 (Bruine de Bruin, 2020; Salari et al., 2020; Swinford et al., 2020), and because people with higher education might have more resources to cope with stress and economic issues. Indeed, several recent studies (Ellett et al., 2003; Taylor et al., 2008; Westerhof and Keyes, 2010; Cheng et al., 2014; Bruine de Bruin, 2020; Hyland et al., 2020; Lopes and Jaspal, 2020; Qiu et al., 2020; Salari et al., 2020; Wang et al., 2020a,b) have found that young people and less educated people have experienced more COVID-related psychological distress than older people and better-educated people. We analyzed the DASS data with the expectation that they would provide a replication of the age and education effects.

## Materials and Methods

### Participants

Overall, 1,506 individuals (from 37 countries) completed the survey. We restricted the analyses to Canadian (*n* = 942) and American (*n* = 273) respondents because we intended to investigate the pandemic at its early stage, and the pandemic had a different time course in different countries. In addition, the majority of the respondents were from Canada (62.5%) and the US (18.1%). The demographic characteristics of this Canada–U.S. sample are reported in Table 1.

**Table 1:** Demographic characteristics of North American sample (*N* = 1,215).

Demographic variable	Statistics
**Age (*M*, *SD*)**	40.17 (15.83)
**Gender** (*n*, %)	
Female	930 (76.5%)
Male	272 (22.4%)
Other	13 (1.1%)
**Education level** (*n*, %)	
Less than high school	9 (0.7%)
Highschool or equivalent	212 (17.4%)
Associate	113 (9.3%)
Undergraduate	394 (32.4%)
Graduate or above	487 (40.1%)
**Job** (*n*, %)	
Job loss	187 (15.4%)
No job loss	1,028 (84.6%)

The factor of relocation was not included for analyses due to a small number of respondents in the relocated group (*n* = 87, 6.7%).

### Materials

#### Transitional Impact Scale (COVID-TIS)

We used a modified version of the TIS-12 (Svob et al., 2014), the COVID-TIS, to assess the type and degree of change brought about by the COVID-19 pandemic. We modified the original scale in two ways: First, we replaced “this event” in all the statements with “COVID-19 pandemic.” Second, we removed two items, “This event has changed where I live,” and “This event has impacted me psychologically.” The first was dropped because respondents were asked the following question at the end of the survey: “Did you move from one residence to another as a direct consequence of the COVID-19 pandemic?” We removed the second item from the TIS because we were using a separate psychological measure, a 21-item DASS scale, to assess specific mental health consequences of the pandemic. The final COVID-TIS scale consists of 10 items (see Table 2); five items load on a material-change subscale, and five on a psychological-change subscale. Participants rated their agreement with each statement on a 1 (*strongly-disagree*)-to-5 (*strongly agree*) scale. The pandemic’s overall material impact was calculated by averaging the ratings of the five material items and its overall psychological impact was calculated by averaging the ratings of the five psychological items.

**Table 2:** Average ratings on COVID-TIS and infection-concern (self and others) from 1 (Strongly Disagree) to 5 (Strongly Agree), and sub-scores of depression, anxiety, and stress scale (DASS) produced by participants with job loss (*n* = 187) and no job loss (*n* = 1,028).

	Job loss		No job loss		Overall	
	*M*	95% *CI*	*M*	95% *CI*	*M*	95% *CI*
Material subscale^***^	3.20	[3.09, 3.32]	2.94	[2.89, 2.99]	2.98	[2.93, 3.03]
I spend my time in difference places now than I did before the COVID-19 Pandemic.	3.36	[3.14, 3.59]	3.33	[3.23, 3.42]	3.33	[3.25, 3.42]
I own different things now than I did before the COVID-19 Pandemic.	2.01	[1.84, 2.17]	1.98	[1.91, 2.05]	1.98	[1.92, 2.05]
My material circumstances now are different than they were before the COVID-19 Pandemic^***^	3.53	[3.35, 3.71]	2.62	[2.54, 2.71]	2.76	[2.68, 2.84]
The activities I engage in now are different from the ones I engaged in before the COVID-19 Pandemic.	4.08	[3.93, 4.23]	3.93	[3.86, 4.00]	3.95	[3.89, 4.01]
The people I spend time with now are not the same people I spent time with before the COVID-19 Pandemic.	3.04	[2.84, 3.25]	2.84	[2.76, 2.92]	2.87	[2.79, 2.95]
Psychological Subscale^***^	3.40	[3.27, 3.53]	3.08	[3.02, 3.13]	3.13	[3.07, 3.18]
My current attitudes are different than the attitudes I held before the COVID-19 Pandemic^***^	3.68	[3.50, 3.86]	3.31	[3.23, 3.39]	3.37	[3.30, 3.44]
**I think about things differently now than I did before the COVID-19 Pandemic**.^**^	3.98	[3.82, 4.14]	3.67	[3.59, 3.74]	3.71	[3.65, 3.78]
My emotional responses now are different than they were before the COVID-19 Pandemic^**^	3.68	[3.51, 3.86]	3.38	[3.31, 3.46]	3.43	[3.36, 3.50]
**My sense of self now is different than it was before the COVID-19 Pandemic**.^***^	3.39	[3.20, 3.58]	2.92	[2.83, 3.00]	2.99	[2.91, 3.06]
My understanding of right and wrong now is different than it was before the COVID-19 Pandemic.	2.27	[2.09, 2.44]	2.10	[2.03, 2.17]	2.13	[2.06, 2.19]
TIS total^***^	3.30	[3.20, 3.40]	3.01	[2.96, 3.05]	3.05	[3.01, 3.09]
Infection-concern (Self)	3.31	[3.13, 3.49]	3.43	[3.35, 3.50]	3.41	[3.34, 3.48]
Infection-concern (Others)	4.21	[4.06, 4.36]	4.15	[4.09, 4.23]	4.16	[4.10, 4.22]
DASS-Depression^***^	8.99	[8.23, 9.75]	7.37	[7.05, 7.69]	7.62	[7.30, 7.93]
DASS-Anxiety^***^	6.24	[5.60, 6.89]	4.59	[4.32, 4.86]	4.84	[4.58, 5.11]
DASS-Stress^*^	10.02	[9.23, 10.81]	9.07	[8.74, 9.40]	9.22	[8.90, 9.53]

Significant between-group effects are marked with^*^.

* *p* < 0.05

** *p* < 0.01

*** *p* < 0.001.

For the current sample, the internal consistency coefficient of COVID-TIS was 0.76 (Cronbach’s *α*_material_ = 0.60; Cronbach’s *α*_psychological_ = 0.81). Corrected item-total correlation for the TIS scale ranged between 0.30 and 0.60.

#### Depression, Anxiety, and Stress Scale

This 21-item scale consists of three self-report measures and assesses the negative related emotional states of depression, anxiety, and stress (Lovibond and Lovibond, 1996). Each of the three subscales contains seven items. Participants rated each item on a 0 (*did not apply to me at all*)-to-3 (*applied to me very much or most of the time*) scale. For the current sample, the internal-consistency coefficient of the DASS was 0.94 (Cronbach’s *α*_depression_ = 0.90; Cronbach’s *α*_anxiety_ = 0.83; Cronbach’s *α*_stress_ = 0.88). Corrected item-total correlation for the 21-item DASS scale ranged between 0.40 and 0.75.

Also, data were collected to capture the demographic characteristics (e.g., gender, age, education, and residential location). We also asked respondents to indicate whether they had lost their job because of the pandemic.

In two separate questions, participants also rated the infection concerns for themselves (*I am concerned that I might become infected with the novel coronavirus*.) and people they know (*I am concerned that close friends and family members might become infected with the novel coronavirus*.) on a Likert scale ranging from 1 (*strongly disagree*) to 5 (*strongly agree*).

At the end of the survey, respondents were provided with an opportunity to describe how they have been impacted by the pandemic. We mention this for the sake of completeness. However, these open-ended responses are not discussed further in this article.

### Procedure

Only people who were 18 years and above were eligible to participate in the study. A snowball sampling strategy was used during participant recruitment. The online survey was disseminated over academic channels (e.g., institution email lists and websites) and social media. The recruitment advertisement contained an URL link to the questionnaire and participants could take the survey at their own pace. At the end of the survey, participants could choose whether they would take part in a follow-up. Participation was strictly voluntary; respondents were not compensated in any way for their cooperation. Only surveys that were completed in their entirety were included for the analysis. Expedited ethics approval was obtained from the Research Ethics Board of the University of Alberta (Pro00099336).

## Results

### Transitional Impact

Table 2 shows the mean TIS ratings, DASS scores, and infection concern responses for the sample as a whole and presented as a function of job loss. These data make several points. First, at least during its early stage, the pandemic did not appear to have produced a radical change in the lives of most respondents. Overall, the TIS scores were not very high; collapsing over groups, the average for the material TIS was 2.98, 95% *CI* = [2.93, 3.03] and the average psychological TIS was 3.13, 95% *CI* = [3.07, 3.18]. By way of comparison, Shi and Brown (2016) found that emigration from China to Canada produced mean material and psychological TIS scores of 4.52 and 4.05, respectively. Second, as predicted, people who lost their jobs as a result of the pandemic indicated that they had experienced more change than those who did not, and this was true for both material change and psychological change. That being said, except for the generic material-change item (see Table 2), between-group differences on the TIS-material items tended to be small or non-existent. In contrast, except for the right-and-wrong item, the job-loss group provided notably higher ratings on the individual TIS-psychological items than the no-job-loss group. Third, as implied by Cronbach’s *α* and consistent with the types of adjustments required by a lockdown, the pandemic altered some aspects of people’s lives more than others. In particular, the TIS-material ratings indicate that the pandemic affected people’s activities and to a lesser extent changed where they spent their time. These item differences reflect the fact that the lockdown restricted the range of activities people could engage in and the locations they could visit. Finally, we note that the psychological TIS ratings indicated that the pandemic, even in this early stage, affected people’s perceptions, attitudes, emotions, and to some extent their sense of self, but not their sense of right and wrong.^1^

These claims are supported by a set of analyses performed separately on the material TIS responses and psychological TIS responses. In both, we conducted a repeated-measures analysis of covariance (ANCOVA) with job status as the between-subject factor, item (i.e., the individual material and psychological TIS questions) as the within-subject factor, and age and education level as covariates. The ANCOVA on material TIS responses produced a reliable main effects of both job status, *F* (1, 1,213) = 17.16, *p* < 0.001, partial *η*^2^ = 0.01, and item, *F* (4, 4,570) = 25.37, *p* < 0.001, partial *η*^2^ = 0.02, and a reliable item × job status interaction, *F* (4, 4,570) = 12.51, *p* < 0.001, partial *η*^2^ = 0.01. We examined the simple main effects regarding the significant interaction and found a reliable effect of job status on “material circumstances,” *F* (1,1,213) = 73.34, *p* < 0.001, partial *η*^2^ = 0.06. *Post hoc* pairwise comparisons with Bonferroni correction showed that the job-loss group rated the “activities” item the highest and the “things” item the lowest, all *p* < 0.001, except for the difference between ‘places’ and ‘material circumstances’ (*p* = 1.00) and between “places” and “people” (*p* = 0.17). Likewise, the ratings for the no-job-loss group, from the highest to the lowest, were “activities,” “places,” “people,” “material circumstances,” and “things,” all *p* < 0.001. Neither covariate played a significant role in this analysis, *p* > 0.05 for both.

The ANCOVA on psychological TIS responses also produced a reliable main effect of job status, *F* (1, 1,211) = 10.61, *p* = 0.001, partial *η*^2^ = 0.01, and a main effect for item, *F* (4, 4,352) = 37.10, *p* < 0.001, partial *η*^2^ = 0.03. The item × job status interaction, however, was not significant, *F* (4, 4,352) = 1.90, *p* = 0.12, partial *η*^2^ = 0.002. The job-loss group rated higher in each item than the no-job-loss group but job status difference (job-loss vs. no-job-loss) in overall psychological TIS score did not depend on the rating of each item; that being said, the same job status difference would be seen for all psychological TIS items. For both groups, the ratings of each item from the highest to the lowest were “thinking about things,” “emotional responses,” “attitudes,” “the sense of self,” and “right and wrong.” *Post hoc* pairwise comparisons with Bonferroni correction indicate that all between-item differences were reliable (*p* < 0.001) except for the difference between the “emotional responses” item and the “attitudes” item.

The two covariates, age and education level, produced reliable effects on the psychological TIS responses, both *p* < 0.05. When we collapsed across items and divided respondents into a younger group (18–40 years old; *n* = 706), a middle age group (41–60 years old; *n* = 328), and older group (at least 61 years old, *n* = 181), we found that participants in the youngest group reported more psychology change (*M* = 3.23, 95% *CI* = [3.16, 3.30]) than the middle aged (*M* = 3.01, 95% *CI* = [2.91, 3.11]), *p* < 0.05, and older group (*M* = 2.93, 95% *CI* = [2.76, 3.08]), *p* < 0.05; the middle aged and older groups did not differ reliably from one another, *p* = 0.94. When we collapsed across items assigned respondents to groups based on education, we found that respondents who had less than a university/college degree (*n* = 334) reported more psychological change (*M* = 3.30, 95% *CI* = [3.20, 3.40]) than those who had at least finished university/college (*n* = 881, *M* = 3.06, 95% *CI* = [3.00, 3.12]), *p* < 0.001.

### Infection Concern Ratings

Overall, infection concern ratings indicated people were less concerned that they would become infected themselves (*M* = 3.41, 95% *CI* = [3.34, 3.48]) than that their friends and family members would become infected (*M* = 4.16, 95% *CI* = [4.10, 4.22]; see Table 2). This observation was confirmed through a repeated measures ANCOVA, using job status as the between-subject factor, infection concern rating (self vs. others) as the within-subject factor, and age and education level as covariates. The analysis yielded a highly reliable main effect of the item (self vs. other), *F* (1, 1,211) = 96.69, *p* < 0.001, partial *η*^2^ = 0.07. The main effect of job status was not significant, *F* (1, 1,211) = 0.13, *p* = 0.72, partial *η*^2^ < 0.001, but the job status × item interaction was *F* (1, 1,211) = 5.08, *p* = 0.02, partial *η*^2^ = 0.004. Nonetheless, we looked into the simple main effects of the interaction and found no significant effect of job status on the infection-concern items (all *p* > 0.05); both job-loss and no-job-loss group indicated greater infection concern for others than for self (both *p* < 0.001). Also, no reliable effects of age and education level were found for the infection concern items (both *p* > 0.05).

**Figure 1:**
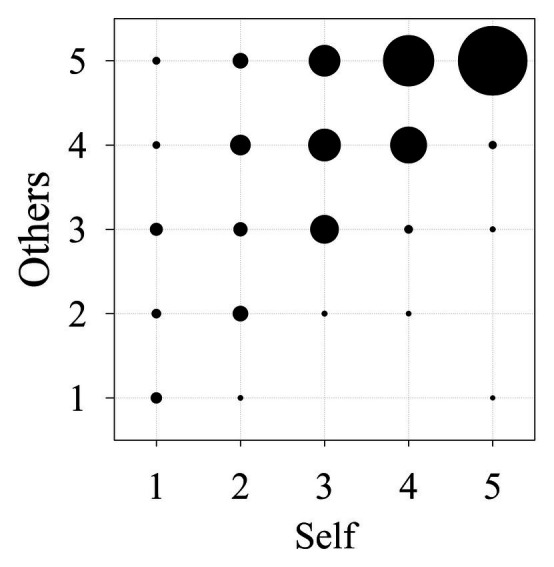
Bubble size indicates percentage of respondents providing a specified pair of ratings for the concern-for-other question (Y-axis) and the concern-for-self question (X-axis).

The bubble plot (see Figure 1) provides a perspective on this finding. The bubbles represent the percentage of respondents that provided a particular pair of ratings (1 = *strongly disagree*; 5 = *strongly agree*) for the self and other infection-concern items. For example, the bubble in the upper right-hand corner represents the percentage of individuals who provided a rating of 5 to both questions. What is striking about these data is how few respondents indicated greater concern for themselves than for others; only 4% of the responses fell below the diagonal (indicating greater concern for self). In contrast, 51% of the responses fell above the diagonal (indicating greater concern for others).

### Depression, Anxiety, and Stress

Mean DASS scores for the job-loss and the no-job-loss groups are presented at the bottom of Table 2. Overall, these scores indicate that individuals who responded to our survey were moderately depressed, mildly anxious, and mildly stressed.^2^ To investigate the effect of job-loss on peoples’ mental health, we ran a multivariate ANCOVA on the ratings of depression, anxiety, and stress, with job status as the fixed factor, and age and education level as covariates. As predicted, and consistent with prior research (Cobb and Kasl, 1977; Dooley and Catalano, 1980; Caplan et al., 1989), respondents in the job-loss group indicated that they were more depressed, *F* (1, 1,211) = 14.67, *p* < 0.001, partial *η*^2^ = 0.01, more anxious, *F* (1, 1,211) = 21.23, *p* < 0.001, partial *η*^2^ = 0.02, and more stressed, *F* (1, 1,211) = 4.68, *p* = 0.03, partial *η*^2^ = 0.004, than respondents in the no-job-loss group.

**Figure 2:**
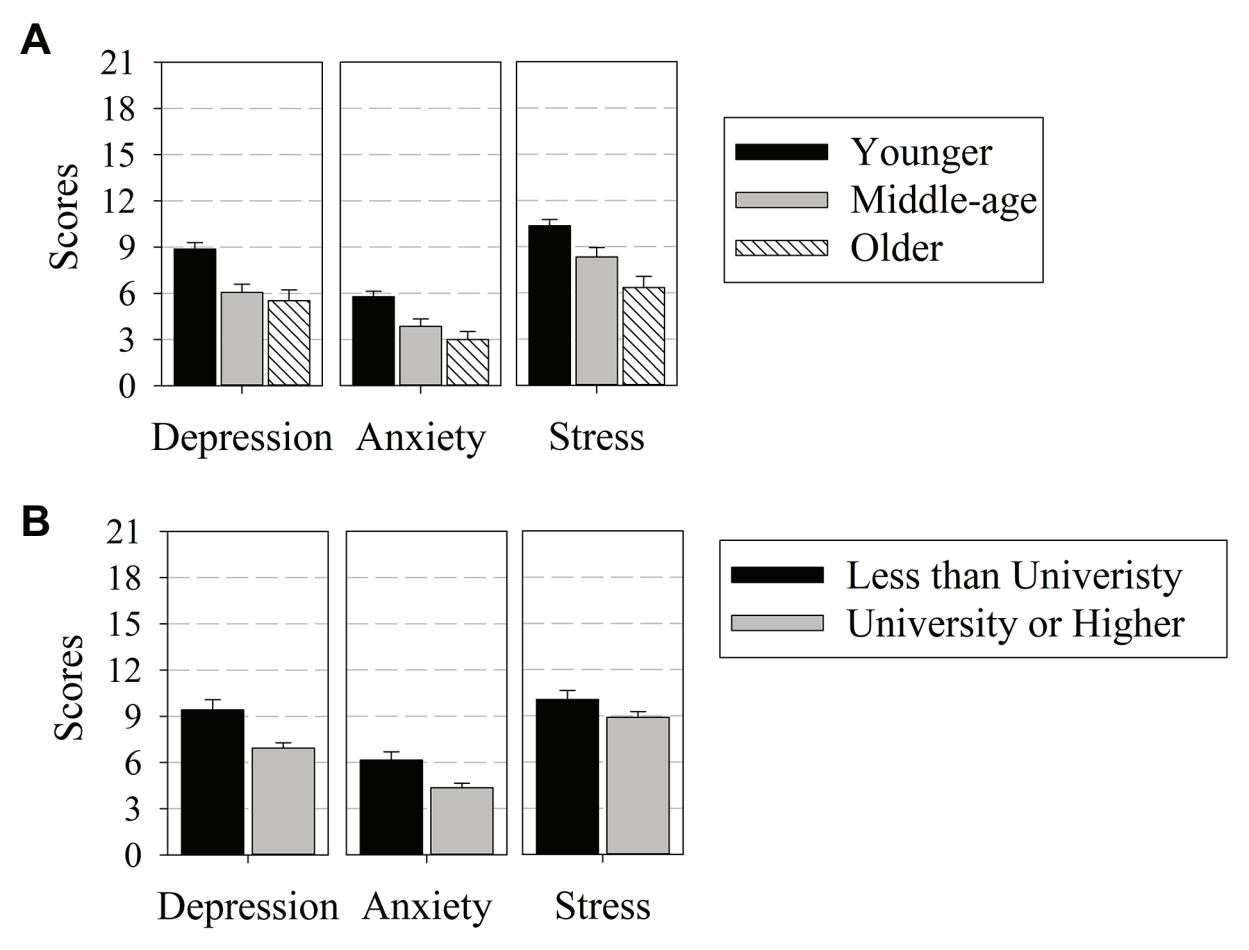
Mean DASS ratings provided by participants of different age groups **(A)** and education level **(B)**. Error bars indicate 95% CIs.

Figure 2 illustrates the effects of age (Panel A) and education level (Panel B) on the three DASS variables. In Panel B, compared to better-educated individuals, less educated individuals were more depressed and more anxious, both *p* < 0.001, but not more stressed, *p* = 0.35. This general pattern replicates prior research (Ellett et al., 2003; Taylor et al., 2008; Lopes and Jaspal, 2020; Wang et al., 2020a,b), Also, as predicted and consistent with prior research (Taylor et al., 2008; Westerhof and Keyes, 2010; Cheng et al., 2014; Bruine de Bruin, 2020; Hyland et al., 2020; Lopes and Jaspal, 2020; Qiu et al., 2020; Salari et al., 2020), we found that younger respondents scored higher on the three DASS subscales than older respondents DASS (Panel A), all *p* < 0.001.

### Predictors of DASS

Table 3 presents a correlation matrix that includes all the variables discussed above. The younger participants tended to have a low level of education, reported greater concerns about their family members and friends, have experienced greater material and psychological changes due to the pandemic and produced higher ratings of depression, anxiety, and stress. With higher education levels, participants indicated less psychological impact and fewer mental problems. Given that many of these correlations were in the moderate range and given our interest in understanding the relation between COVID-related factors and negative mental health outcomes, we conducted a set of regressions, one for each of the three DASS measures. Specifically, for each DASS variable, we fitted a multiple linear regression model, using the full set of available variables – age, education level, COVID-TIS material and psychological ratings, self, and other infection-concern ratings. These variables were entered hierarchically with age and education entered first as control variables. The output of these analyses is presented in Table 4. These regressions indicated that depression and stress were both predicted by material change, psychological change, and concern for others, but not by concern for self. Anxiety was also predicted by psychological change, concern for others, and concern for self, but not by material change.

**Table 3:** Correlation matrix of age, education level, infection-concerns, COVID-TIS, and DASS.

	Education	Concern-self	Concern-others	TIS-material	TIS-psychological	TIS-total	DASS-depression	DASS-anxiety	DASS-stress
Age	0.35^**^	0.04	−0.12^**^	−0.07^*^	−0.15^**^	−0.13^**^	−0.31^**^	−0.26^**^	−0.25^**^
Education		0.002	−0.03	0.003	−0.17^**^	−0.08^**^	−0.22^**^	−0.19^**^	−0.10^**^
Concern-self			0.60^**^	0.16^**^	0.23^**^	0.24^**^	0.12^**^	0.26^**^	0.19^**^
Concern-others				0.17^**^	0.26^**^	0.27^**^	0.23^**^	0.31^**^	0.28^**^
TIS-material					0.32^**^	0.78^**^	0.18^**^	0.21^**^	0.20^**^
TIS-psychological						0.85^**^	0.41^**^	0.47^**^	0.44^**^
TIS-total							0.36^**^	0.42^**^	0.41^**^
DASS-depression								0.63^**^	0.68^**^
DASS-anxiety									0.73^**^

Significant Spearman’s correlation coefficients are marked with ^*^.

* *p* < 0.05

** *p* < 0.01

*** *p* < 0.001.

**Table 4:** Hierarchical regression results for DASS.

	*B*	95% CI		*SE B*	*β*	*R*^2^	∆ *R*^2^	*F*
		Lower bound	Upper bound					
**Depression**						0.24		63.15^***^
*Step 1: control variables (demographic)*							0.11^***^	
Age	−0.07	−0.08	−0.05	0.01	−0.19^***^			
Education	−0.74	−0.99	−0.48	0.13	−0.15^***^			
*Step 2: TIS subscale*							0.12^***^	
Material	0.37	0.01	0.73	0.18	0.05^*^			
Psychological	1.81	1.49	2.13	0.16	0.31^***^			
*Step 3: Infection concern*							0.01^**^	
Self	−0.21	−0.50	0.08	0.15	−0.05^ns^			
Friends and Family	0.61	0.26	0.95	0.18	0.11^**^			
**Anxiety**						0.29		81.28^***^
*Step 1: control variables (demographic)*							0.08^***^	
Age	−0.05	−0.06	−0.03	0.01	−0.16^***^			
Education	−0.48	−0.69	−0.28	0.11	−0.12^***^			
*Step 2: TIS subscale*							0.18^***^	
Material	0.27	−0.03	0.56	0.15	0.05^ns^			
Psychological	1.74	1.48	1.10	0.13	0.36^***^			
*Step 3: Infection concern*							0.03^***^	
Self	0.43	0.20	0.66	0.12	0.11^***^			
Friends and Family	0.40	0.12	0.68	0.14	0.09^**^			
**Stress**						0.28		69.79^***^
*Step 1: control variables (demographic)*							0.07^***^	
Age	−0.07	−0.10	−0.05	0.01	−0.20^***^			
Education	−0.04	−0.30	0.21	0.13	−0.01^ns^			
*Step 2: TIS subscale*							0.17^***^	
Material	0.39	0.03	0.75	0.18	0.06^*^			
Psychological	2.12	1.80	2.43	0.16	0.36^***^			
*Step 3: Infection concern*							0.02^***^	
Self	0.11	−0.18	0.40	0.15	0.02^ns^			
Friends and Family	0.63	0.29	0.98	0.18	0.12^***^			

* *p* < 0.05

** *p* < 0.01

*** *p* < 0.001.

ns not significant.

## Discussion

This study examined the transitional impact of the COVID-19 pandemic and its effect on people’s mental health during the initial stage of the outbreak in North America (i.e., Canada and US). As predicted, people who lost their job due to the pandemic experienced a greater change in their material and psychological condition, and higher levels of depression, anxiety, and stress than those who did not. Moreover, younger adults were more depressed, anxious, and stressed compared to middle-aged and older adults. Likewise, less well-educated people indicated that they were more troubled by the pandemic than better-educated people. Surprisingly, people showed more infection concern for their family and friends than for themselves, regardless of their job status, age, or level of education. Material and psychological change, and infection concern for close others were associated with depression and stress while anxiety was associated with psychological change and concern for both self and others contracting the and infection. These findings raise two interesting questions: one concerns the relationship between the transitional impact of the pandemic and the distress it appears to have caused, and the other concerns the strong tendency for people to rate their concern for others higher than their concern for themselves. We take up these questions below.

First, it is clear from the TIS data that the pandemic, in its early stage, did not produce a marked change in people’s material circumstances. In other words, at the time, people were not dealing with calamitous changes to their living situation.^3^ Yet, we found elevated levels of depression, anxiety, and stress and a relatively strong link between these measures of psychological distress and the degree of psychological change caused by the pandemic. Perhaps, the simplest way to explain this pattern is to recognize that the pandemic has engendered a great deal of uncertainty and uneasiness about the future (McGinty et al., 2020; Zandifar and Badrfam, 2020) and to assume that this type of uncertainty can have a negative impact on people’s mental health and their worldview (Torales et al., 2020). Consistent with this position, we found that the young, the unemployed, and the under-educated – groups with the least financial security – experienced the most psychological distress and reported the most psychological change. Going forward, at a minimum, we expect that current levels of psychological distress will persist for the duration of the pandemic. We can also predict that the level of psychological distress will increase sharply when programs like Canada Emergency Response Benefit (CERB), Canada Emergency Student Benefit (CESB), and Unemployment Insurance, Coronavirus Aid, Relief, and Economic Security (CARES), and Coronavirus Relief Fund (CRF) in the US are defunded and evictions, foreclosures, and bankruptcies become more common (Bloomberg Opinion Editorial Board, 2020; Goodman, 2020; Irwin, 2020).^4^

As in for the infection concern findings, we believe that there are two factors at play. First, people were likely to believe that they can control, to some degree, the risks they would take on. However, they might recognize that they cannot control the risk-taking behavior of others (Choi et al., 2020; Korajlija and Jokic-Begic, 2020). Therefore, they are less concerned for themselves than they are for others in their circle. Second, recall that respondents were asked to consider “close friends and family members” when rating the concern-for-others item; it seems likely that most people know people who fall into one of the high-risk categories (e.g., people in their 70s or older, people with pulmonary issues, etc.; Swinford et al., 2020). If the concern-for-other response is anchored by the status of the most vulnerable person in a person’s social network, it follows that the concern-for-other responses should, on average, be higher than the concern-for-self responses.

### Limitation

Due to the time-sensitive nature of the COVID-19 outbreak, we adopted a convenient sampling strategy. As a result, there was an oversampling of a certain network of peers (e.g., students and academics), leading to selection bias. Thus, caution is required when generalizing these findings, particularly the aggregate means. That being said, we have reason to believe that the relational findings (i.e., the strong correlations between the Psychological TIS scores and the DASS scores) would be generalized to a representative sample and as we have noted throughout the presentation of these data, a number of our findings are consistent with those reported by other research teams (e.g., the relation between age and depression). We also take a note that only age and education covariates have been included in the analysis and there might be other covariates such as- socioeconomic status, gender etc., that could have had a role on explaining the outcome. Finally, it would be useful, going forward, to collect data that would allow us to test the hypothesis that it is uncertainty about the future, rather than (or in addition to) changes in one’s current living situation, which accounts for the COVID-related increases in depression, anxiety, and stress.

## Conclusion

Looking back at the onset of the COVID-19 pandemic, we remember the early days of lockdown as a time of change and emotional upheaval. Yet our data paint a somewhat different picture. It is true, many people experienced a change in their routines, but these changes were not typically life-altering. Likewise, people reported elevated levels of psychological distress, but not extreme levels of psychological distress. The picture may well have been different had we been able to focus on hot spots (e.g., metropolitan New York) or particularly vulnerable populations (e.g., frontline healthcare workers). Nonetheless, we believe that it is important to recognize that there is often a gap, sometimes a very wide gap, between our immediate emotional response to a crisis and the way that crisis affects our lives (Brown et al., 2009; Brown and Lee, 2010). Therefore, we believe that it will be interesting and useful to follow the pandemic over time as some people habituate to a set of relatively minor adjustments to their routines and others contend with devastating life changes.

## Data Availability Statement

The raw data supporting the conclusions of this article will be made available after the authors have completed a multi-wave data collection protocol and have published their findings.

## Ethics Statement

The studies involving human participants were reviewed and approved by Research Ethics Board, University of Alberta (Pro00099336). Written informed consent for participation was not required for this study in accordance with the national legislation and the institutional requirements.

## Author Contributions

EH and NB were involved in developing the study design. EH oversaw data collection. The analysis was done by EH, LS, and NB. Manuscript preparation was done by EH, LS, and NB. All authors contributed to the article and approved the submitted version.

### Conflict of Interest

The authors declare that the research was conducted in the absence of any commercial or financial relationships that could be construed as a potential conflict of interest.

## Footnotes

^1^“The right-and-wrong” item is included to assess the impact of a target event on a personal ethical/moral believes, which is a part of people’s psychological element.

^2^According to the manual for 21-item DASS (Lovibond and Lovibond, 1996), the cut-off score for normal, mild, and moderate depression are 0–4, 5–6, and 7–10, respectively. For anxiety the cut-offs are 0–3, 4–5, 6–7 representing normal, mild, and moderate; and for stress it is 0–7, 8–9, and 10–12 defining normal, mild, and moderate individually.

^3^When the survey was conducted in late March 2020, Canadians and Americans were being cushioned against the negative impact of the Pandemic by policies that provided economic support for the unemployed and deferred rent and mortgage payments.

^4^In terms of the measures collected in this present study, defunding federal financial support programs should produce a sustainably increase in material TIS ratings, the psychological TIS ratings and the DASS scores. It should also cause the two TIS scores to be more highly correlated. Importantly, the Material TIS rating should become a robust predictor of the DASS, indicating that the people are reacting to the negative consequences of their changing live circumstances rather than to their concerns about an uncertain future.
